# Non‐motor role of the cerebellum: Cerebellar mutism syndrome in a child with a small hemorrhagic contusion in the dentate nucleus: A case report and literature review

**DOI:** 10.1002/ccr3.9375

**Published:** 2024-08-28

**Authors:** Moayad Ahmed, Mukashfi Ali, Alaaeldin Ginawi

**Affiliations:** ^1^ Department of Neurosurgery Aliaa Specialist Hospital Khartoum Sudan; ^2^ Nottingham University Hospital Nottingham UK

**Keywords:** cerebellar cognitive affective syndrome, cerebellar mutism syndrome, computed tomography of the brain, Glasgow coma scale

## Abstract

**Key Clinical Message:**

Our case report highlights that Prompt recognition of cerebellar mutism syndrome (CMS) is critical in clinical practice, as it is often misdiagnosed as just being reduction in the level of consciousness, particularly in pediatrics trauma patients.

**Abstract:**

Cerebellar mutism syndrome is defined as transient mutism following posterior fossa surgery, hemorrhage or traumatic insults involving the cerebellum. Cerebellar mutism syndrome (CMS) is now recognized as a form of cerebellar cognitive affective syndrome (CCAS/Schmahmann syndrome). CMS following head injury is exceedingly rare with very few cases reported. Five years old boy with normal developmental milestones and no significant medical history, sustained closed head injury following road traffic accident, the clinical scenario in addition to the radiological findings led to the diagnosis of cerebellar mutism syndrome. CMS is defined as of neurologic and cognitive disorders, mainly speech disorder. Patient non‐motor signs recovered in a period of few weeks from the mutism syndrome with remaining mild motor deficit. CMS is a set of neurologic and cognitive disorders, the most distinct of which is speech disorder (usually reversible), what is unique about this case is the selective site of the contusion at the region of the dentate nucleus and superior cerebellar peduncle. Such cases offer a better understanding to the function of the cerebellum and its role in cognition. Additionally, the knowledge of this aspect of cerebellar function helps in better predicting the clinical course of such lesions which in turn helps in communication and explanation to patient's families.

## 1 INTRODUCTION

Cerebellar mutism syndrome is defined as transient mutism following posterior fossa surgery, hemorrhage or traumatic insults involving the cerebellum, the mutism usually associated with emotional liability, ataxia, hypotonia, and irritability. The historical understanding of cerebellar mutism syndrome (CMS) has unfolded over time. While the initial components, such as mutism and ataxia, were described by Bailey in 1939,^15^ subsequent reports by Cairns (1948) and Daly and Love documented cases of “akinetic mutism” following posterior fossa surgery.^11^ Guidetti & Fraioli in 1975 also described almost similar case in their series of dentate nucleus stereotactic lesioning^12^ Notably, Rekate coined the term “cerebellar mutism” itself in 1985.^1^ Now cerebellar mutism syndrome is considered a form of what is known as cerebellar cognitive affective syndrome (CCAS/Schmahmann's syndrome).^4^ The exact mechanism behind this entity is not well understood but involvement of cerebellar pathways, regions, and circuits have been proposed; the concept of dysmetria of thought has now been used to explain the non‐motor aspect of cerebellar lesions.^6^ Diagnosing CMS require high index of suspicion, patient usually present with low GCS which can result in misdiagnosis. Careful neurologic and psychological assessment in addition to neurologic imaging would help in reaching the diagnosis.

## 2 CASE HISTORY/EXAMINATION

Five years old boy with free medical background and normal developmental milestones involved in a road traffic accident (pedestrian hit by car). There was a history of immediate loss of consciousness followed by convulsions several episodes each lasting for a few seconds after which patient regained consciousness but was irritable and excessively crying. No other neurologic events. Patient admitted to another hospital then transferred to our institute.

On initial physical examination, patient looked irritable and was crying. The vital signs were all within the normal ranges. There were multiple small lacerations over the forehead and the left elbow. Glasgow coma scale (GCS) 11/15 E.4 V.2 M.5, Pupils were equal and reactive to light bilaterally, upper and lower limbs on the left side were moving less, hypotonic and hyporeflexic, (power was unassailable due to low level of consciousness), and there was no cranial nerves involvement. Physical examination of other systems was unremarkable. Patient admitted to the pediatrics high dependency unit (HDU) and treated conservatively with neurologic assessment and observation. He was mute for the entire period, but on day 8 of admission, patient started to obey commands and started to say words (one word). Patient discharged home on Day 16 of admission in good condition with Glasgow coma scale 15/15, the right side was normal, but there was hypotonia, hyporeflexia, and PG 4 on the left side, signs of cerebellar dysfunction were present on the left. Patient was followed up regularly in the outpatient clinic for 1 year.

## 3 METHODS (DIFFERENTIAL DIAGNOSIS, INVESTIGATIONS, AND TREATMENT)

**FIGURE 1:**
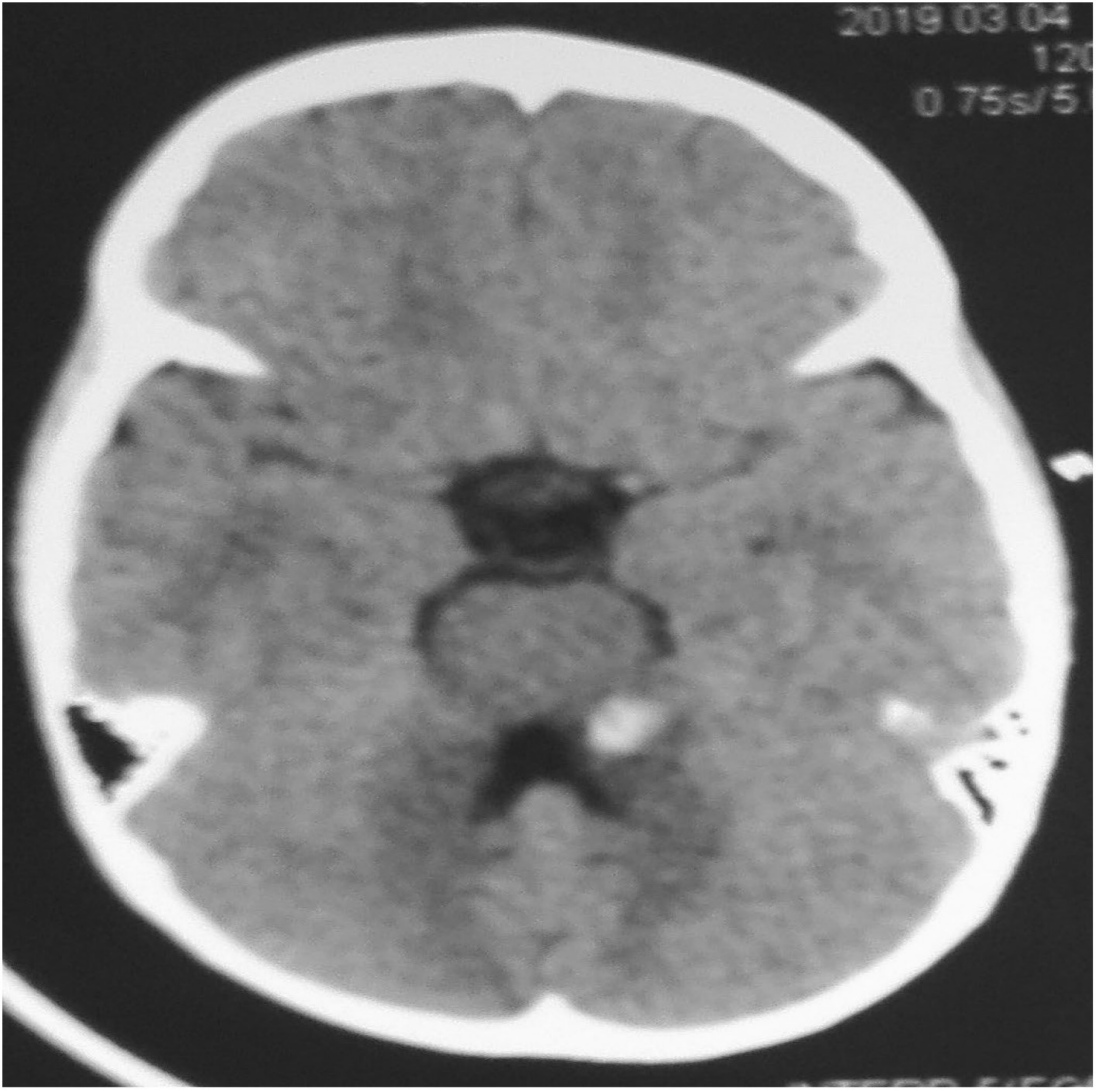
Non‐contrast CT brain axial slice performed at the initial presentation. The CT shows hyperdense lesion (resembling hemorrhage/hematoma) seen in the region of the left superior peduncle and dentate nucleus. The reminder of the scan was unremarkable.

**FIGURE 2:**
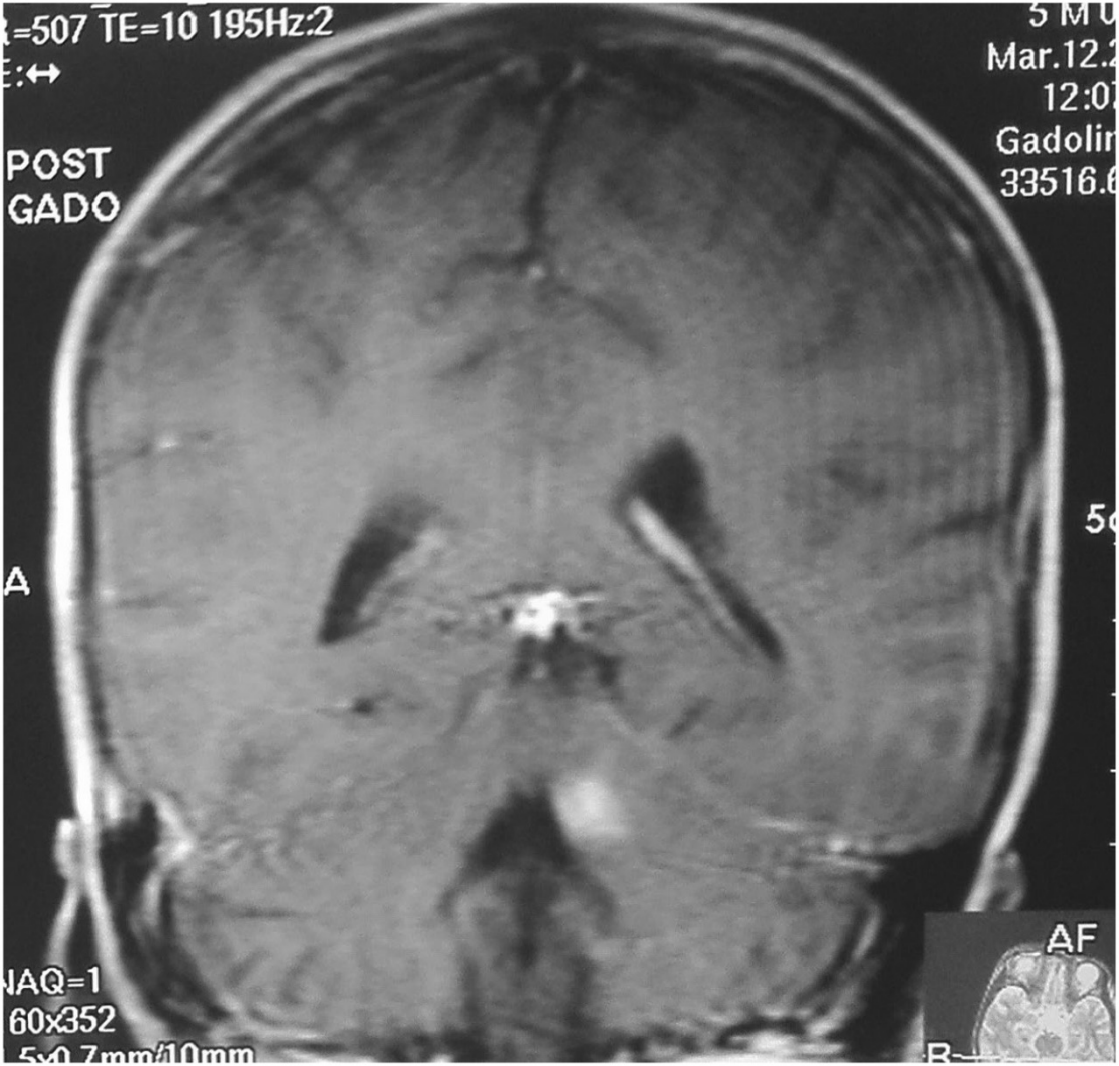
Post contrast MRI of the brain, T1 sequence coronal slice. The scan re‐demonstrates the same lesion seen on the initial CT.

**FIGURE 3:**
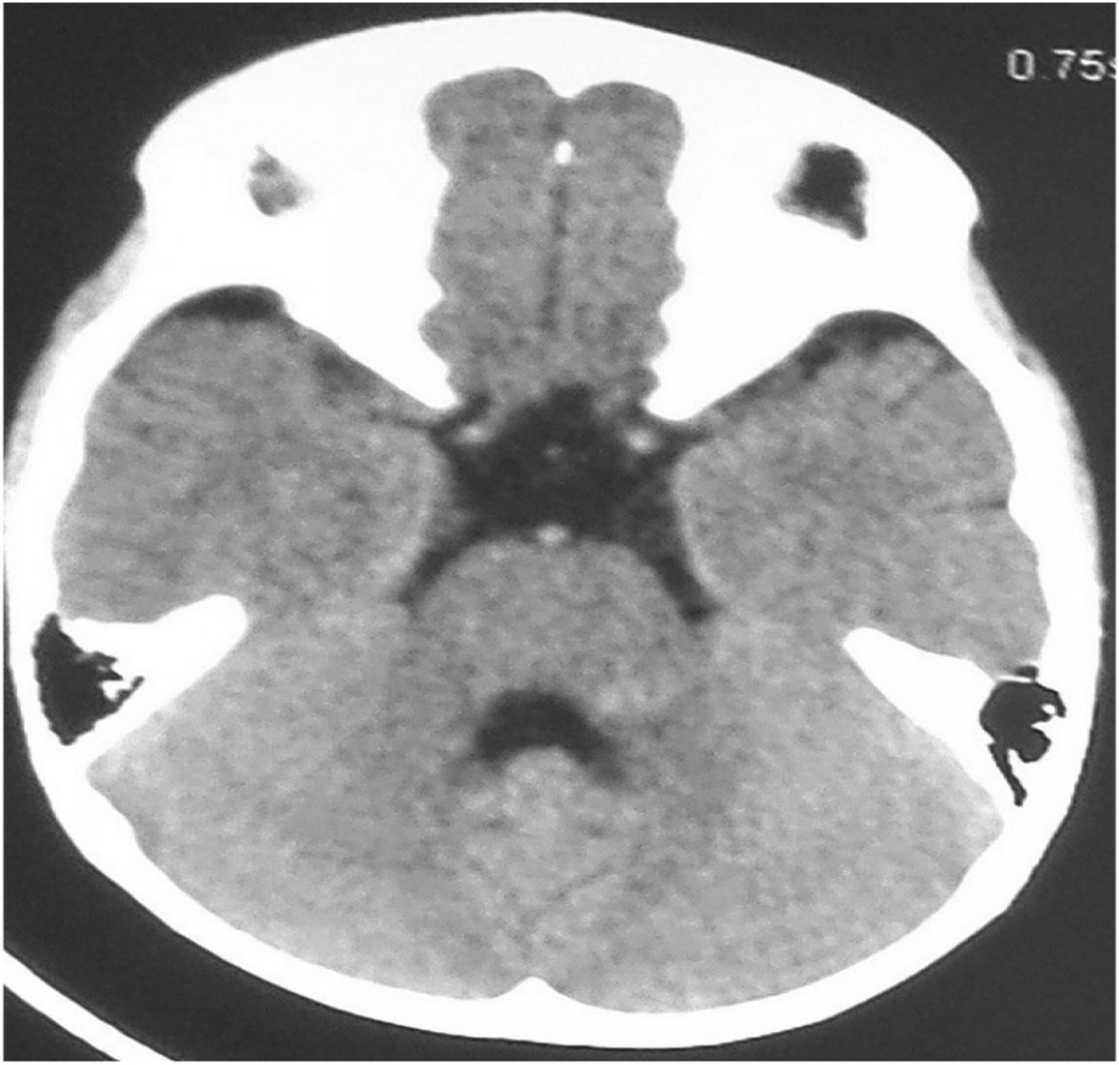
Non‐contrast CT brain, axial slice performed 7 days after the initial CT showing complete resolution of the abnormality.

Blood Investigations showed no abnormalities. Initial CT scan of the head on admission showed small hemorrhagic contusion on the left superior cerebellar peduncle and dentate nucleus (Figure 1) and MRI was done few days later to show the exact site of the contusion and to exclude any other pathology rendering patient mute (Figure 2). Seven days after admission follow‐up CT brain was done and showed resolving hematoma (Figure 3) in conjunction with patient clinical improvement. Patient admitted to the pediatrics high dependency unit (HDU) and treated conservatively with neurologic assessment and observation.

## 4 CONCLUSION AND RESULTS

Since CMS is typically seen postoperatively in patients with posterior fossa tumors, awareness of the condition may be limited for experts in the trauma setting. Understanding the condition will help in providing better care to patients and reassurance to their families. In everyday practice such diagnosis can be overlooked and attributed to the decreased level of consciousness especially in the pediatrics population. Good understanding of the role of the cerebellum in cognition, affect and language is crucial as it will help the clinicians to communicate with patients and families and to provide the best plan of treatment management plan.

## 5 DISCUSSION

Mutism is defined as the loss of speech while maintaining the ability to hear and comprehend language/speech. On the other hand, aphasia is defined as loss of the ability to comprehend language and/or loss of the ability to formulate language.^1^ In the case of cerebellar mutism syndrome, there is transient mutism following cerebellar insults. CMS is a well‐described condition occurring in the pediatric population following posterior fossa surgery, particularly where a vermian incision is made.^3^ Two cases reported by Yokota et al. described traumatic cerebellar injury complicated by a traumatic medial longitudinal fasciculus (MLF) syndrome or cerebellar mutism.^5^ Other case reports are reviewed in Table 1. Cerebellar mutism syndrome is considered a form of cerebellar cognitive affective syndrome (CCAS/Schmahmann's syndrome).^4^ CCAS is defined as impairment in cognitive domains of executive function, spatial cognition, and language and affect (feelings, emotions, and mood) following cerebellar insults. Albazron and colleagues stated that their findings raise the possibility that among patients with CCAS, there may be some locations injuries which related to absence or presence of cerebellar mutism. These areas include cerebellar midline structures such as vermis and the fastigial nuclei, which are almost injured in every patient develop mutism, on other hand, patients with CCAS without mutism were mostly has lesions lateral to the mildline structures like the interposed, dentate nuclei, and left superior cerebellar peduncle.^13^ Many cerebello‐cerebral pathways have been identified connecting relevant cognitive areas of the cerebral cortex to the cerebellum. These areas include the superior temporal gyrus (language area), parahipppocampal areas (spatial memory), prefrontal region (working memory, complex reasoning, and judgment), and the limbic system (feelings, emotions, and mood)^2^, ^3^ the concept of dysmetria of thought is comparable to that of dysmetria of movement resulting from cerebellar insults.^4^ Some investigators showed that some functions are lateralized in the cerebellum in association with that of the cerebral cortex, one example is language, if the language is dominant in frontal lobe of the left hemisphere, the right side of the cerebellum will be responsible for language.^2^ Other studies found that right cerebellar hemisphere lesions results in greater cognitive deficits than the left cerebellar hemisphere lesions, also one study found that left cerebellar hemisphere is more involved in visuospatial functions.^6^ On the other hand, the deep cerebellar nuclei were shown to have specific functions, for example, the dentate nucleus involved in cognition, the interpositus nucleus involved in motor function, and the fastigial involved in limbic function.^3^ Beside the motor functions, the cerebellum regulate emotions, anxiety, and affect. Chen et al. found that suppression of cerebellar Purkinje cells firing has resulted in rapid excitation of certain forebrain areas that influenced these previous functions.^14^ A study found that the freezing behavior found in many animals when faced by predators is meditated by interactions between periaqueductal gray (PAG) and the cerebellum which it fine‐tune this act.^17^ PAG in association to the amygdala and thalamus has also been implicated in vocalization suppression when the animal is approached by a predator,^16^ this complex circuits between cerebellum, thalamus, and the amygdala may play a crucial rule in the pathophysiology of this phenomena. Reviewing the literature regarding the post‐traumatic CMS also revealed and concluded that all cases were in the pediatric population. Cerebellar hemisphere and vermian areas were the common sites of the insult which was mainly a closed head injury. Maximum recovery time from onset to near complete regain of cognitive functions was 8–9 weeks, recovery itself started few days post trauma with no comprehensive neurocognitive assessment made during or post recovery period for all cases.

**TABLE 1:** Literature review of cases reported of post‐traumatic cerebellar mutism.

Authors	Year	Gender	Age	Mode of trauma	Radiologic findings	Mutism signs start time	Recovery time
Yokota et al.^5^	1990	Male	6 years	RTA	Injury to cerebellar vermis or left cerebellar hemisphere	Not mentioned	Unknown
Erashin et al.^7^	1997	Male	2.5 years		Hematoma in the right paravermian region	Not mentioned	2 months
Koh et al.^8^	1997	N/A	N/A	RTA	Left cerebellar hemisphere (small contusion) and left cerebellar peduncle (small focal hemorrhage)	3–5 days post trauma	8 weeks
Fujisawa et al.^2^	2005	Male	7 years	RTA	ASDH in posterior fossa	After hematoma evacuation	39 days
Kariyattil et al.^9^	2014	Male	6 years	RTA	Contusion of the cerebellar vermis and left cerebellar hemisphere	Since admission	1 month
Lahirish et al^10^	2021	Female	8 years	Penetrating head injury	Skull base fracture through the floor of the posterior cranial fossa in association with right cerebellar contusion and edema	4 days post trauma	1 month

Abbreviations: ASDH, acute subdural hematoma; RTA, road traffic accident.

## AUTHOR CONTRIBUTIONS

**Moayad Ahmed:** Formal analysis; investigation; project administration; supervision. **Mukashfi Ali:** Conceptualization; writing – original draft. **Alaaeldin Ginawi:** Investigation.

## FUNDING INFORMATION

No funding was received for this research.

## CONFLICT OF INTEREST STATEMENT

The author declares that they have no conflicts of interest.

## CONSENT

Written informed consent was obtained from the patient to publish this report in accordance with the journal's patient consent policy.

## Data Availability

The data that support the findings of this study are available from the corresponding author upon reasonable request.
